# Mesencephalic basolateral domain specification is dependent on Sonic Hedgehog

**DOI:** 10.3389/fnana.2015.00012

**Published:** 2015-02-17

**Authors:** Jesus E. Martinez-Lopez, Juan A. Moreno-Bravo, M. Pilar Madrigal, Salvador Martinez, Eduardo Puelles

**Affiliations:** ^1^Instituto de Neurociencias de Alicante, Universidad Miguel Hernandez, Consejo Superior de Investigaciones Científicas (UMH-CSIC)Alicante, Spain; ^2^Instituto Murciano de Investigacion Biomedica IMIB-Arrixaca (CIBERSAM)Murcia, Spain

**Keywords:** midbrain, *Shh*, *Barhl1*, *Nhlh1*, *Six3*, basolateral domain, alar-basal boundary

## Abstract

In the study of central nervous system morphogenesis, the identification of new molecular markers allows us to identify domains along the antero-posterior and dorso-ventral (DV) axes. In the past years, the alar and basal plates of the midbrain have been divided into different domains. The precise location of the alar-basal boundary is still under discussion. We have identified *Barhl1, Nhlh1* and *Six3* as appropriate molecular markers to the adjacent domains of this transition. The description of their expression patterns and the contribution to the different mesencephalic populations corroborated their role in the specification of these domains. We studied the influence of *Sonic Hedgehog* on these markers and therefore on the specification of these territories. The lack of this morphogen produced severe alterations in the expression pattern of *Barhl1* and *Nhlh1* with consequent misspecification of the basolateral (BL) domain. *Six3* expression was apparently unaffected, however its distribution changed leading to altered basal domains. In this study we confirmed the localization of the alar-basal boundary dorsal to the BL domain and demonstrated that the development of the BL domain highly depends on *Shh*.

## Introduction

**Figure 1:**
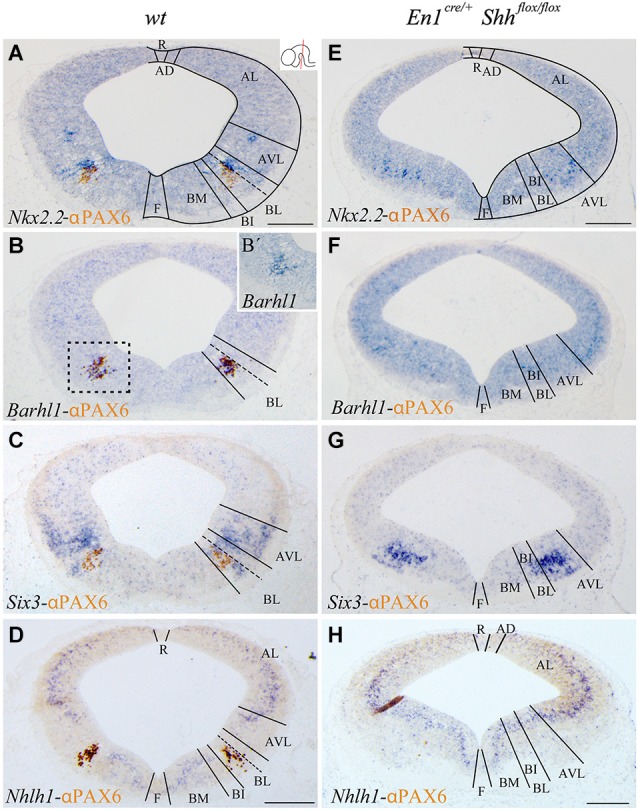
***Barhl1, Six3* and *Nhlh1* expression pattern along the midbrain DV axis**. Coronal mesencephalic sections of wild type **(A–D)** and *En1^cre/+^*; Shh^flox/flox^ **(E–H)** embryos at E12.5 processed by immunochemistry and *in situ* hybridization **(A–H)**. Schematic subdivision from dorsal to ventral using *Nkx2.2* and PAX6 as reference **(A)**. The *Barhl1* expression was restricted to the ventral region of the BL domain **(B)**. Square in B shows magnification of this area before immunochemistry **(B’)**. The *Six3* expression included the entire AVL and BL domains **(C)**. The *Nhlh1* expression was exclusively negative for the BL domain **(D)**. In the conditional mutant, the whole basal plate showed a reduction in size. *Nkx2.2* was detected only in the AVL and it was completely lost, as PAX6, in the BL/BI domains **(E)**. *Barhl1* expression was also lost in the BL/BI domains **(F)**. *Six3* expression was more compacted in the BL/BI and AVL domains **(G)**. The expression of* Nhlh1* was continuous along the DV axis, being also positive for the altered BL/BI domains **(H)**. Inset in A shows the level of the sections. Abbreviations: AD, alar dorsal; AL, alar lateral; AVL, alar ventrolateral; BI, basal intermediate; BL, basolateral; BM, basal medial; F, floor; R, roof. Scale bar = 200 μm.

The midbrain, located between the forebrain and the hindbrain, is subdivided, along the antero-posterior axis, into two mesomeres (m1–m2) (Hidalgo-Sánchez et al., 2005; Moreno-Bravo et al., 2012; Puelles et al., 2012). The rostral mesomere (m1) includes the tectal gray and the superior and inferior colliculi among other populations. The caudal mesomere is rather small and corresponds to the pre-isthmic region (Puelles et al., 2012). Along the dorso-ventral axis (DV) the midbrain is subdivided into roof plate, alar plate, basal plate and floor plate. This DV patterning is due to the effect of two opposing organizing regions, the roof plate and the floor plate. These regions secrete signaling molecules establishing gradients, e.g., *Wnt1* and *BMP* from the roof plate and *Shh* from the floor plate. These gradients are translated by neuroblasts into positional information. This triggers differentiation programs that specify different neuronal populations along the DV axis (Basler et al., 1993; Dickinson et al., 1995; Lee and Jessell, 1999; Patten and Placzek, 2000; Chizhikov and Millen, 2005; Placzek and Briscoe, 2005; Ingham and Placzek, 2006; Szabó et al., 2009a,b). In the midbrain this DV organization is referred to as tectum (dorsal) and tegmentum (ventral), which acquire their alar and basal characteristics under the influence of roof and floor plate organizers respectively. These alar and basal regions are subsequently subdivided into smaller domains based on different gene expression patterns (Nakatani et al., 2007; Kala et al., 2009; Moreno-Bravo et al., 2012; Puelles et al., 2012). The alar plate is compartmentalized in alar dorsal (AD), alar lateral (AL) and alar ventrolateral (AVL) domains, whereas the basal plate is divided into basolateral (BL), basal intermediate (BI) and basal medial (BM) domains (Nakatani et al., 2007; Kala et al., 2009; Moreno-Bravo et al., 2012; Puelles et al., 2012; see Figure 1A). The BL domain is an intricate transition region extending ventrally form the alar-basal boundary into the basal plate, which displays a major morphological complexity. The whole BL domain expressing *Nkx2.2* can be further subdivided into dorsal (dBL) and ventral (vBL) subdomains. The dBL is *Gata2* positive and gives rise to GABAergic neurons and the vBL is *Pax6* positive and gives rise to glutamatergic neurons (Kala et al., 2009). The fact that this domain generates different neuronal subtypes highlights the complexity of this area.

It has been reported that the basal midbrain exhibits distinct phenotypes depending on the *Shh* signaling alteration level. Loss of function mutations generates a complete ablation of floor and basal plate neural structures (Chiang et al., 1996; Fogel et al., 2008). A specific conditional inactivation in the *En1* expression domain (midbrain and rhombomere 1) leads to a reduction in dopaminergic neurons, a disorganization of the red nucleus (RN) and a complete loss of the oculomotor complex (III) among other abnormalities (Perez-Balaguer et al., 2009).

The aim of this work is to describe new markers that could serve as tools to analyze and describe the basal and alar domains surrounding the alar-basal boundary. We chose *Barhl1, Nhlh1* and *Six3* for this detailed characterization and also studied possible roles in *Shh* signaling using a *Shh* conditional inactivation driven by *En1*. We selected *Barhl1, Nhlh1* and *Six3*, based on gene expression databases as Eurexpress^1^ and Allen Brain Atlas,^2^ because they are specifically expressed in basal mesencephalic compartments among other territories. These transcription factors have proven useful tools for the characterization of basal midbrain organization and have helped to understand the phenotypic alterations of the *Shh* conditional mutant. We described their expression pattern in both E12.5 and E15.5. At E12.5 the DV domains are being generated, the neuronal precursors are still proliferating in the ventricular area and much of them are migrating to their final position. At E15.5, the basal midbrain is completely stablished and the nuclei are already visible.

## Materials and methods

### Animals

The conditional *Shh* mutant mouse contains the *Shh^tm2AMC^* allele where the exon 2 is flanked by LoxP sequences as previously reported (Lewis et al., 2001). The *En1^cre/+^* transgenic line was previously described (Kimmel et al., 2000). The conditional mutant embryos were generated crossing double heterozygous males (*En1^cre/+^*; Shh^flox/+^) with homozygous females (*Shh^flox/flox^*) as previously described (Perez-Balaguer et al., 2009). The day when the vaginal plug was detected was considered as embryonic day 0.5 (E0.5). Embryos were fixed in phosphate buffer solution (PBS 1x, NaCl 13 mM, Sigma S3014; KCl 0.3 mM, Sigma P9541; Na_2_HPO_4_ 1 mM, Sigma S3264 and KH_2_PO_4_ 0.2 mM, Sigma P9791) with 4% paraformaldehyde (PFA, Panreac 141451.1211) overnight at 4°C and cryoprotected for storage at −20°C. Embryos were washed twice in 100% butanol (Panreac 14.682.1211) previously to be wax embedded (Gemcut emerald paraffin, Catalog no. 24364-1) and then sectioned in parallel series (7 μm thick). A total of 20 pregnant females were used. Each experiment was triplicated to confirm the result. All mouse experiments were performed according to protocols approved by the Universidad Miguel Hernandez CEIE committee.

### *In situ* hybridization

The sections were dewaxed at 65°C and completely rehydrated. To facilitate probe penetration, tissue was incubated during 4 min with proteinase K (0.1 mg/ml) in PBS-T (PBS 1x with 0.1% Tween 20, Sigma P1379) and post fixed in 4% PFA. The sections were washed in PBS-T and prehybridized for 1 h in hybridization buffer comprised of 50% deionized formamide (Amresco, 0606), SALT 1X (NaCl 0.2 M, Sigma S3014, tris-HCl 9 mM Sigma T3253, Tris-Base 1 mM, Sigma T6066, NaH_2_PO_4_·2H_2_O 5 mM, Scharlau SO0334, Na_2_HPO_4_ 5 mM, Sigma S3264 and EDTA 5 mM, Sigma E5134) Denharts 2X (Bio Basic Canada D0062), Dextran sulfate 0.2 mM (Amresco, 0198) and 0.1% tRNA (Sigma R6625). The RNA probes were obtained from Source Bioscience/ ImaGenes (*Barhl1*, IRAVp968G10116D; *Nhlh1*, IRAVp968D02101D) or constructions kindly provided by Dr. J. Rubenstein (*Nkx2.2*) and Dr. L. Puelles (*Six3*). These digoxigenin-labeled RNA probes (DIG-11-UTP, Roche Diagnostics, 11209256910) were denaturalized at 80°C and incubated with the tissue in hybridization buffer overnight at 62°C. The next day sections were washed in wash solution with 50% SSC 1x pH 7.0 (NaCl 0.15 M, Sigma S3014 and Na_3_C_6_H_5_O_7_·2H_2_O 15 mM, Sigma C8532), 25% formamide (Sigma, F7503) and 0.1% tween 20 at 65°C and incubated with MABT 1x pH 7.4 (NaCl 40 mM, maleic acid 20 mM, NaOH 40 mM and 0.1% tween 20) with 10% sheep serum (Sigma, S2263) and 20% blocking reagent (Roche, 10057177103). After blocking, tissue was incubated overnight at 4°C in the same solution with an alkaline phosphatase-coupled anti-digoxigenin antibody (1:3500, Roche, 11093274910). Excess of non-specific anti-digoxigenin antibody was extensively washed in MABT. Finally, the sections were washed with NTMT (NaCl 0.1 M, Sigma S3014, Tris-HCl 0.1 M pH 9.5, Sigma T3253, MgCl2·6H_2_O 0.05 M, VWR 1.05833 and 0.1% tween-20) and incubated overnight at room temperature in NTMT with 0.45 μl/ml of 4-Nitro blue tetrazolium chloride (NBT, 75 mg/ml, Roche, 70227721) and 3.5 μl/ml of 5-Bromo-4-Chloro-3-indolyl-phosphate, (BCIP, 50 mg/ml, Roche 11585002001). The NBT/BCIP was used for the colorimetric reaction to detect the presence of the hybridized probes. The alkaline phosphatase reacts with these substrates and produces a solid blue precipitate.

### Immunohistochemistry

The sections were dewaxed, completely rehydrated and for antigen retrieval boiled in sodium citrate 0.1 M pH 6. The sections were washed in PBS-T and incubated in PBS-T with 1.5% H_2_O_2_ for 30 min to inactivate endogenous peroxidase. After inactivation, tissue was washed in PBT and blocked 1 h in PBS-T with 0.1% albumin bovine serum (BSA, A2153, Sigma) and 10% lysine 1 M (L5626, Sigma). Next, sections were incubated overnight at room temperature in PBS-T with 0.1% BSA and 0.01% sodium azide (S2002, Sigma) with αNKX2.2 (1:5, raised in mouse, Hybridoma Bank, 74.5A5) or αPAX6 (1:200, raised in rabbit, Covance, PRB-278P). The day after, the tissue was rinsed in PBS-T and incubated 1 h with αMouse (1:200, raised in goat, Vector Labs, BA-2020) or αRabbit (1:200, raised in goat, Vector Labs, BA-1000) biotinylated secondary antibody. Afterwards, the sections were washed in PBS-T and incubated in PBS-T with Avidin–Biotin Complex (1:500, Vectastain PK-4000) for 1 h. Finally, tissue was washed in PBS-T and Tris 0.1 M pH 7 and the immunolabeling was revealed in Tris 0.1 M with 1% 3-3′ diaminobenzidine tetrahydroc (DAB, Acros Organics W0572M) and 0.003% H_2_O_2_ leading to a brown precipitate (0.025% Ammonium Nickel Sulphate was added to obtain black precipitate).

## Results

### Role of *Shh* in the development of DV mesencephalic domains

With the aim to better characterize the different domains of the basal midbrain, we studied the expression pattern of *Barhl1, Nhlh1* and *Six3*. These markers have been proven to be useful for a better understanding of the complexity of the basal midbrain, specifically in the BL domain. We used *Nkx2.2* and PAX6 as a well-known reference for a precise localization of these markers. At E12.5 *Nkx2.2* delimited the BL domain and a small area in the AVL whereas PAX6 was only restricted to the vBL domain (Figure 1A). *In situ* hybridization for the new markers showed a discrete expression of *Barhl1* in the vBL, overlapping with the PAX6 positive domain (Figures 1B,B’). *Six3* displayed a complex expression pattern in the alar-basal transition including the AVL and BL domain (Figure 1C). *Nhlh1* was widely expressed along the DV axis of the midbrain, including all the domains of the alar region, the BM and BI domains in the basal plate and the floor plate. Only the BL domain was negative for this marker (Figure 1D).

After the characterization of the precise localization of our markers, our aim was to investigate the influence of the basal organizer in the specification of the BL domain. We used the conditional mutant *En1^cre/+^; Shh^flox/flox^* to abolish the expression of *Shh* in the midbrain. This strain allows us to investigate the effect of the loss of *Shh* on the expression pattern of *Barhl1, Nhlh1* and *Six3*. The entire basal plate showed a complete disorganization and the expression of our markers presented the following alterations. At E12.5, *Nkx2.2* expression was only detected in the AVL domain. Both *Nkx2.2* and PAX6 were completely lost in the BL domain (Figure 1E). *Barhl1*, that colocalized with PAX6, was also absent at E12.5 (Figure 1F). *Six3* expression was maintained in the alar-basal transition, but the expression domain was more compact (Figure 1G), probably due to the incorrect specification of the basal territory that receives the *Six3* positive neurons. *Nhlh1* expression also displayed clear alterations. The discontinuity of expression found in the BL domain disappeared in the conditional mutant (Figure 1H).

**Figure 2:**
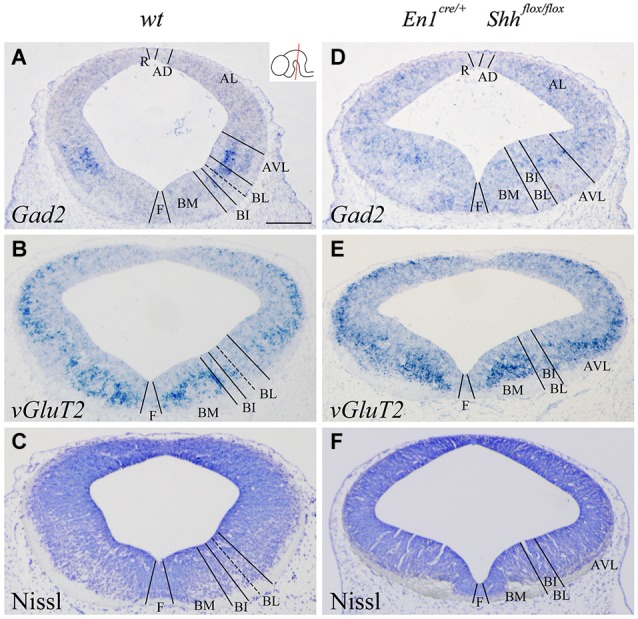
**Alterations of the neuronal precursors in the absence of *Shh***. Coronal mesencephalic sections of wild type **(A–C)** and *En1^cre/+^*; Shh^flox/flox^ **(D–F)** embryos at E12.5 processed by *in situ* hybridization **(A,B,D,E)** and basic Nissl staining **(C,F)**. Neuronal markers for GABAergic **(A)** and glutamatergic **(B)** neurons showed their contribution to the DV domains. The absence of *Shh* showed the altered development of both GABAergic **(D)** and glutamatercig **(E)** precursors. Basic Nissl staining showed the abnormal developing of the basal plate when compared with control **(C,F)**. Inset in A shows the level of the sections. Abbreviations: AD, alar dorsal; AL, alar lateral; AVL, alar ventrolateral; BI, basal intermediate; BL, basolateral; BM, basal medial; F, floor; R, roof. Scale bar = 200 μm.

We confirmed the morphological interpretation of the expression patterns by comparison with *Gad2* and *vGluT2* distribution and basic Nissl staining. The distribution of GABAergic (Figures 2A,D) and glutamatergic neurons (Figures 2B,E) showed an altered distribution in the conditional mutant when compared with control. In the basic Nissl staining, we verified the abnormal development of the DV domains described above (Figures 2C,F).

We conclude that *Barhl1* is a useful marker for the vBL; whereas *Six3* covers the AVL and the BL domains and *Nhlh1* could serve as a negative marker for the BL as it labels all the other domains. The changes in expression pattern in the conditional mutant indicate a clear defect in the specification of the BL domain in the absence of the morphogen *Shh*.

### Differentiation of midbrain neuronal populations in the altered basal domains

**Figure 3:**
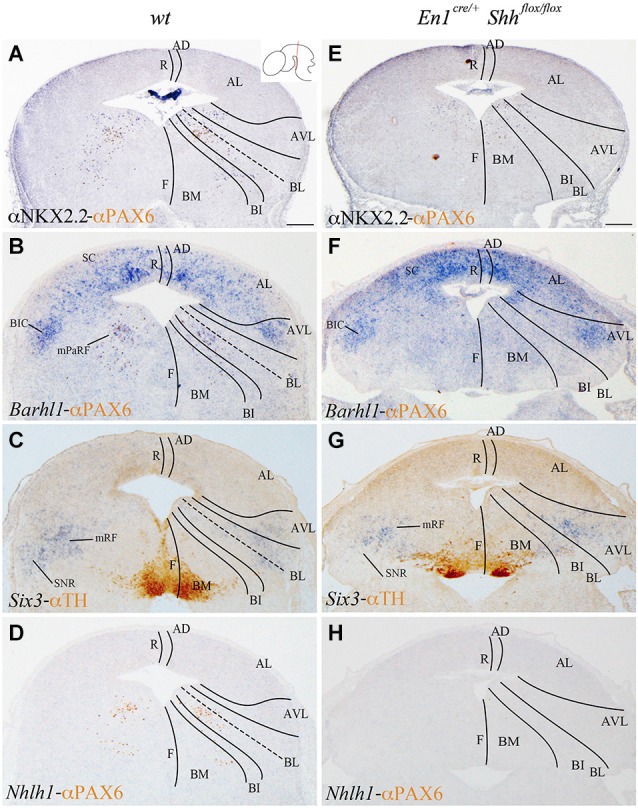
**Contribution of *Barhl1, Six3* and *Nhlh1* to the basal populations**. Coronal mesencephalic sections of wild type **(A–D)** and *En1^cre/+^*; Shh^flox/flox^ **(E–F)** embryos at E15.5 processed by immunochemistry **(A,E)** or immunochemistry and *in situ* hybridization **(B–D,F–H)**. Schematic subdivision from dorsal to ventral using *Nkx2.2* and PAX6 as reference **(A)**. The expression pattern of *Barhl1* showed its contribution to the whole alar plate and the mPaRF in the basal plate **(B)**. The expression pattern of *Six3* showed its contribution to the mRF and SNR (limited by TH positive neurons; **(C)**. *Nhlh1* was downregulated at this stage **(D)**. The absence of *Shh* caused a reduction of *Nkx2.2* **(E)** and the lost of PAX6 and *Barhl1* **(F)** in the basal midbrain.* Six3* expression presented a scattered and wider distribution in the mRF **(G)**. *Nhlh1* was not expressed as in the wild type **(H)**. Inset in A shows the level of the sections. Abbreviations: AD, alar dorsal; AL, alar lateral; AVL, alar ventrolateral; BI, basal intermediate; BIC, bed nucleus of the brachium of the inferior colliculus; BL, basolateral; BM, basal medial; F, floor; mPaRF, mesencephalic pararubral formation; mRF, mesencephalic reticular formation; R, roof; SC, superior colliculus; SNC, Substantia nigra pars compacta; SNR, Substantia nigra pars reticulata. Scale bar = 200 μm.

We analyzed the expression pattern of these markers at E15.5 to associate the domains observed at E12.5 with differentiated neuronal populations. *Nkx2.2* and PAX6 expression was used again as a reference. *Nkx2.2* was still detected in the AVL and BL domains, but migrated cells were also located in the mantle layer of the BI and BM domains. PAX6 labeled the vBL domain and a few migrated scattered cells were detected in the BI and BM domains (Figure 3A). *Barhl1* extended its expression to the alar plate, including the superior colliculus (SC) and the bed nucleus of the brachium of the inferior colliculus (BIC) in the AVL (Figure 3B). In the vBL, coincident with the location of PAX6 positive neurons (compare Figures 3B,D), *Barhl1* defined a discrete population lateral to the oculomotor complex that is part of the pararubral formation (mPaRF, Figure 3B). Expression of *Six3* was observed in the mantle layer of the AVL, BL, BI and BM. It corresponded to the mesencephalic reticular formation (mRF) of the AVL, BL and BI. *Six3* positive cells located in the BM correspond with tangentially migrating neurons forming the Substantia nigra pars reticulata (SNR; Figure 3C), the population located lateral to the dopaminergic Substantia nigra pars compacta (Figure 3C). At this stage, *Nhlh1* expression was completely downregulated (Figure 3D).

**Figure 4:**
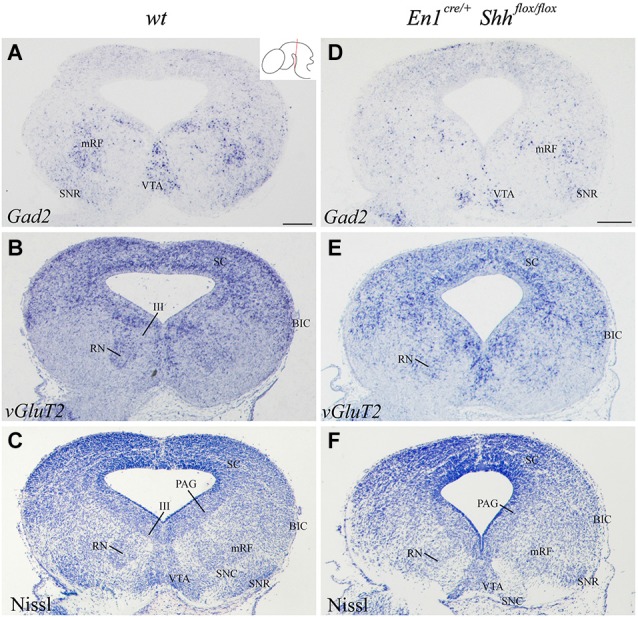
**Alteration of the basal population in the absence of *Shh***. Coronal mesencephalic sections of wild type **(A–C)** and *En1^cre/+^*; Shh^flox/flox^ **(D–F)** embryos at E15.5 processed by *in situ* hybridization **(A,B,D,E)** and basic Nissl staining **(C,F)**. The absence of *Shh* affected the GABAergic **(A,D)** and glutamatergic **(B,E)** populations, here verified by Nissl staining **(C,F)**. Inset in A shows the level of the sections. Abbreviations: III, oculomotor complex; BIC, bed nucleus of the brachium of the inferior colliculus; mRF, mesencephalic reticular formation; PAG, periaqueductal gray; RN, red nucleus; SC, superior colliculus; SNC, Substantia nigra pars compacta; SNR, Substantia nigra pars reticulate; VTA, ventral tegmental area. Scale bar = 200 μm.

In order to further investigate the effect of loss of *Shh* on the differentiation of the neuronal populations we also studied the expression of these markers at E15.5. *Nkx2.2* expression was almost absent in the conditional mutant; we detected a small number of positive neurons in the mantle layer of the AVL, BI/BL and BM domains (Figure 3E). PAX6 positive neurons were absent (Figures 3E,F,H). *Barhl1* expression in the alar plate was apparently normal. It was present in the SC and BIC (Figure 3F). In contrast, its expression in the basal plate was completely lost (Figure 3F). The expression of *Six3* in the mantle layer barely differed from the wild type expression. In the conditional mutant the expression pattern was similar but more compact (Figure 3G). *Nhlh1* was not expressed as it was described previously in the control (Figure 3H). The misspecification observed in the BI and BL domains were corroborated by the study of *Gad2* and *vGluT2* distribution and basic Nissl staining. The distribution of GABAergic neurons was clearly altered. The three populations described, mRF, SNR and ventral tegmental area (VTA) presented a strong reduction in the number of neurons (Figures 4A,D). The glutamatergic neurons did not display obvious changes. Nevertheless, the RN displayed a clear alteration in its neuronal distribution, occupying a wider territory (Figures 4B,E). This phenotype was confirmed by the analysis of basic Nissl staining (Figures 4C,F).

In summary, our markers allowed us to follow the development of all the DV domains at late embryonic stages and we were able to identify neuronal subpopulations that were generated in each of these domains. Additionally, we demonstrated that *Shh* is required for the proper specification of the mesencephalic BL domain. Its absence results in alterations of this domain causing the loss or miss-expression of *Nkx2.2, Pax6, Barhl1, Nhlh1* and *Six3* leading to an incorrect differentiation of neuronal populations located in this territory.

## Discussion

### New markers for the DV axis and their contribution to the mesencephalic neuronal population

**Figure 5:**
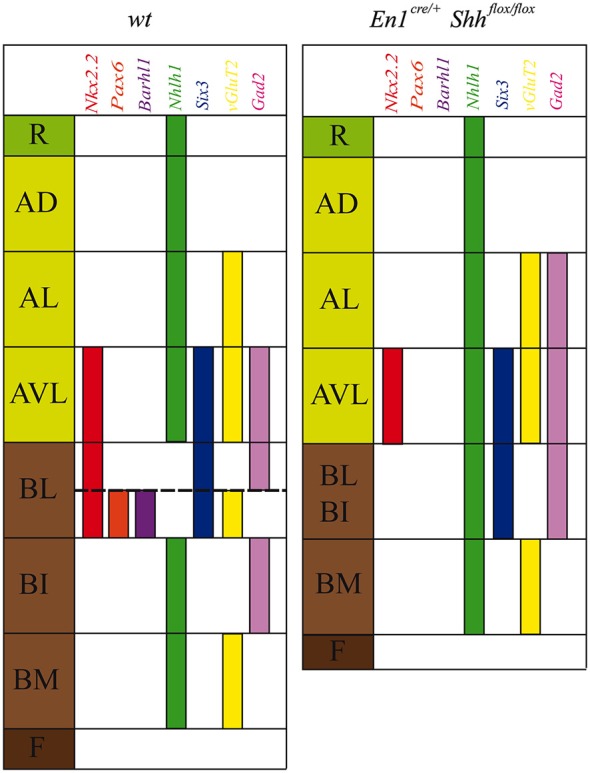
**Schematic representation of DV mesencephalic domains**. Model of the DV patterning in the midbrain domains at E12.5 in the wild type and the conditional mutant. The lack of *Shh* generated an altered development of the basal plate and a miss-expression of *Nkx2.2, Pax6, Barhl1, Nhlh1, Six3, vGluT2* and *Gad2*.

The study of gene expression patterns along the DV axis of the midbrain helped us to further characterize the alar and basal plate subdivisions (Nakatani et al., 2007; Kala et al., 2009; Puelles et al., 2012). In the present work we have identified *Barhl1, Nhlh1* and *Six3* as useful markers for a detailed characterization of these subdivisions (Figure 5). The *Barhl1* expression pattern, at E12.5, specifically defined the vBL. It mimics the distribution of the PAX6 protein. Both markers allowed us to describe two distinct subdomains within the BL midbrain region (Figure 5). One subdomain located in the dBL is negative for these markers and gives rise to GABAergic neurons. The second subdomain located in the vBL is *Barhl1* and *Pax6* positive and generates glutamatergic neurons (Kala et al., 2009; Figure 5). At E15.5, *Barhl1* also presented an expression domain in the alar plate defining the SC and the BIC, while in the basal plate it defined a discrete population close to the oculomotor complex. *Barhl1* and *Pax6* have been implicated in the specification of other glutamatergic populations. In the cerebellum *Pax6*, expressed in migrating granular cells, is implicated in the proper migration of these neurons (Engelkamp et al., 1999). In the same region, the expression of *Barhl1* coincides with *Pax6*. However, there is no implication for a functional interaction. The current opinion is that they work independently (Landsberg et al., 2005). In the cerebellum, *Barhl1* is also involved in the generation of the glutamatergic granular cells, but is involved in a pathway that controls the migration and survival of their progenitors (Bulfone et al., 2000; Li et al., 2004; Lopes et al., 2006; Rachidi and Lopes, 2006). At E12.5, the expression of *Barhl1* as occurs in the cerebellum, should be related to the proper migration and survival of the glutamatergic neurons generated in the vBL. At E15.5, *Barhl1* in the alar plate is involved in the maintenance and survival, but not in the differentiation of the SC neurons (Li and Xiang, 2006).

At E12.5, *Six3* labeled the domains adjacent to the alar-basal boundary, defining both the AVL and the BL domains (Figure 5). In the BL, there was a clear difference between the vBL and the dBL, showing a stronger expression in the latter. This transcription factor is a well-known marker for the GABAergic lineage, expressed in neuronal precursors after the onset of *Gata2* expression (Virolainen et al., 2012). The GABAergic nature of the AVL and dBL (Kala et al., 2009) indicates a role of *Six3* in the development of these neuronal populations. At E15.5 its expression extended into the mantle layer covering almost all the GABAergic mRF, the pararubral nucleus and the SNR (Paxinos and Watson, 1998; Paxinos et al., 2009; Moreno-Bravo et al., 2012; Madrigal et al., 2015).

Finally, *Nhlh1* expression at E12.5 served as a negative marker for the BL domain, as it is the only negative domain along the DV axis (Figure 5). Unfortunately, at E15.5, its expression was completely down regulated. This early *Nhlh1* expression has been related to a possible role in promoting migration or in preventing postmitotic cells from re-entering the cell cycle erroneously (Li et al., 1999; Schmid et al., 2007).

In summary, we have identified a new set of genetic markers for the characterization of mesencephalic DV domains at both sides of the alar-basal boundary. They additionally allowed us to identify neuronal populations generated in these domains.

### The basolateral domain of the midbrain depends on *Shh* for its specification

*Shh* is secreted from the ventricular layer of the floor and basal plates and has an important role in the development of mesencephalic basal populations (Hynes et al., 1995; Litingtung and Chiang, 2000; Agarwala and Ragsdale, 2002; Abeliovich and Hammond, 2007; Joksimovic et al., 2009; Perez-Balaguer et al., 2009). Little is known about possible roles in the specification of the domains adjacent to the alar-basal boundary. In order to unveil if *Barhl1, Nhlh1* and *Six3* are regulated by *Shh* we used the conditional mutant *En1^cre/+^*; Shh^flox/flox^. The basal plate of this mutant is strongly affected in the BM domain, the III is lost, the SNC and the VTA are strongly reduced and the RN is altered (Hynes et al., 1995; Abeliovich and Hammond, 2007; Joksimovic et al., 2009; Perez-Balaguer et al., 2009). The analysis of *Barhl1, Nhlh1* and *Six3* finally allows us to better describe these defects in the basal midbrain. This was complex until today due to the reduced number of specific markers for the BI and BL. One of the most evident defects was the loss of BL domain identity where all of the markers were affected (Figure 5). At E12.5, *Barhl1* expression in the vBL domain was absent. This coincides with the loss of *Pax6* expression in the same domain (Perez-Balaguer et al., 2009). At E15.5, *Barhl1* was expressed normally in the alar plate, since the absence of *Shh* does not affect the specification of the alar plate (Echevarría et al., 2003; Chizhikov and Millen, 2005; Vieira et al., 2010; Martinez-Ferre and Martinez, 2012; Martinez-Ferre et al., 2013). However, a small *Barhl1* positive population located close to the oculomotor complex was completely lost. It is plausible to hypothesize that this population originated from the *Barhl1* positive vBL domain described at E12.5. *Six3* expression at E12.5, in the absence of *Shh*, was similar to the expression pattern observed in controls. Nevertheless, its pattern appeared more compact probably due to a general reduction in the size of this domain. At E15.5, *Six3* expression continued without apparent alterations. Nevertheless, *Six3* positive neurons located in the mantle layer were disorganized. This anomalous distribution could be due to a general alteration of the mRF in the mesencephalic basal plate. However, the absence of *Shh* does not affect *Six3* expression itself. A possible explanation for that could be that the fate of GABAergic neurons has already been determined by *Gata2*, which is expressed before the onset of *Six3* expression in the basal midbrain (Virolainen et al., 2012). The fact, that GABAergic nuclei that populate the basal midbrain arise from *Shh* negative ventricular domains, further supports this hypothesis (Joksimovic et al., 2009; Madrigal et al., 2015). Finally, *Nhlh1* functions as a negative marker for the BL domain at E12.5. In the absence of *Shh*, it is expressed along the entire DV axis including the altered BL domain, suggesting again a loss of identity of this area.

In conclusion, our data corroborate the strong influence of *Shh* on the specification of the most dorsal domain of the mesencephalic basal plate, the BL domain. This strong *Shh* dependence allowed us to validate its basal identity. Therefore, the dorsal limit of the BL domain help us to precisely locate the alar-basal boundary; all the domains ventral to this boundary are *Shh* dependents.

## Author contributions

All authors had full access to all the data in the study and take responsibility for the integrity of the data and the accuracy of the data analysis. Conceived and designed the experiments: Jesus E. Martinez-Lopez, Salvador Martinez and Eduardo Puelles; Performed the experiments: Jesus E. Martinez-Lopez, Juan A. Moreno-Bravo and M. Pilar Madrigal; Analyzed the data: Jesus E. Martinez-Lopez and Eduardo Puelles; Wrote the article: Jesus E. Martinez-Lopez and Eduardo Puelles; Obtained funding: Salvador Martinez and Eduardo Puelles.

## Conflict of interest statement

The authors declare that the research was conducted in the absence of any commercial or financial relationships that could be construed as a potential conflict of interest.

## Footnotes

^1^http://www.eurexpress.org/

^2^http://www.brain-map.org/
